# Gold(III) to Ruthenium(III) Metal Exchange in Dithiocarbamato Complexes Tunes Their Biological Mode of Action for Cytotoxicity in Cancer Cells

**DOI:** 10.3390/molecules26134073

**Published:** 2021-07-03

**Authors:** Maria Dalla Pozza, Christophe Orvain, Leonardo Brustolin, Nicolò Pettenuzzo, Chiara Nardon, Christian Gaiddon, Dolores Fregona

**Affiliations:** 1 Department of Chemical Sciences, University of Padova, Via F. Marzolo 1, 35131 Padova, Italy; maria.dalla-pozza@chimieparistech.psl.eu (M.D.P.); leo.brus90@gmail.com (L.B.); nicolo.pettenuzzo.np@gmail.com (N.P.); chiara.nardon@unipd.it (C.N.); 2 Interface Recherche Fondamentale en Cancérologie, Université de Strasbourg, Inserm UMR_S 1113, 3 av. Molière, 67200 Strasbourg, France; orvain@unistra.fr

**Keywords:** gastric cancer, metal complexes, chemotherapy, dithiocarbamates, drug mechanism of action, ER stress, p53, autophagy

## Abstract

Malignant tumors have affected the human being since the pharaoh period, but in the last century the incidence of this disease has increased due to a large number of risk factors, including deleterious lifestyle habits (i.e., smoking) and the higher longevity. Many efforts have been spent in the last decades on achieving an early stage diagnosis of cancer, and more effective cures, leading to a decline in age-standardized cancer mortality rates. In the last years, our research groups have developed new metal-based complexes, with the aim to obtain a better selectivity for cancer cells and less side effects than the clinically established reference drug cisplatin. This work is focused on four novel Au(III) and Ru(III) complexes that share the piperidine dithiocarbamato (pipe-DTC) as the ligand, in a different molar ratio. The compounds [AuCl_2_(pipeDTC)], [Au(pipeDTC)_2_]Cl, [Ru(pipeDTC)_3_] and β-[Ru_2_(pipeDTC)_5_] have been synthesized and fully characterized by several chemical analyses. We have then investigated their biological properties in two different cell lines, namely, AGS (gastric adenocarcinoma) and HCT116 (colon carcinomas), showing significant differences among the four compounds. First, the two gold-based compounds and β-[Ru_2_(pipeDTC)_5_] display IC_50_ in the µM range, significantly lower than cisplatin. Second, we showed that [AuCl_2_(pipeDTC)] and β-[Ru_2_(pipeDTC)_5_]Cl drive different molecular mechanisms. The first was able to induce the protein level of the DNA damage response factor p53 and the autophagy protein p62, in contrast to the second that induced the ATF4 protein level, but repressed p62 expression. This study highlights that the biological activity of different complexes bringing the same organic ligand depends on the electronic and structural properties of the metal, which are able to fine tune the biological properties, giving us precious information that can help to design more selective anticancer drugs.

## 1. Introduction

Nowadays, cancer represents the second most important cause of death and morbidity in Europe, after cardiovascular diseases [1]. In this context, the most used and effective small-molecule anticancer agent is cisplatin, which was serendipitously discovered back in 1965, by Barnett Rosenberg and Loretta Van Camp [2]. Despite its efficacy against many types of cancers, such as testicular, ovarian and bladder neoplasia, this anticancer agent presents several severe side effects, including gastrointestinal symptoms, renal tubular damage, and ototoxicity. Those side effects illustrate the lack of selectivity of cisplatin for cancer cells, and cause a reduced therapeutic window, which represents the major inconveniences of the applicability and efficacy of cisplatin [3,4]. These serious limitations, along with the intrinsic or acquired resistance of some types of tumors to cisplatin [5], have prompted researchers to design new platinum-based drugs with better pharmacological efficacy and fewer side effects. Carboplatin and oxaliplatin are approved worldwide as the second-generation platinum(II) drugs [6], and satraplatin was been the first platinum(IV) complex to reach phase III clinical trials. Unfortunately, these compounds have also displayed side effects and resistance mechanisms [7].

For these reasons, in the last decades researchers have focused on the design and development of novel metal complexes using different transition metals, with the aim to improve the effectiveness and diminish the side effects of platinum-based drugs. Among the non-platinum antitumor agents, several gold(III) and ruthenium(III) complexes gained particular interest because of their strong inhibitory activity on cancer cell growth [8,9,10]. In addition, the immunosuppressive and anti-inflammatory properties of the gold ion itself [11] have made gold(III) complexes exciting for testing as anticancer compounds since the immune tumoral microenvironment became a target of anticancer therapy. On the other hand, ruthenium(III) complexes present several interesting aspects, such as (i) the rate of ligand exchange comparable to that of Pt(II) compounds [12], (ii) multiple and accessible oxidation states [13,14], and (iii) the ability to mimic iron in the physiological environment [15,16], which make this metal particularly interesting in the development of anticancer agents.

In this context, in the last decades, our research group has been designing some gold(III)– and ruthenium(III) dithiocarbamato complexes [17,18]. Dithiocarbamates (DTC) have not accidentally been chosen as ligands; in fact, some dithiocarbamato salts have displayed good efficacy as chemo-protectants during cisplatin treatment, decreasing its nephrotoxicity without changing its antineoplastic activity [19]. Indeed, this class of compounds could diminish the interactions between the metal center and the sulfur-containing biomolecules, which are generally believed to have negative effects on the therapeutic efficacy of platinum-based drugs [20,21]. Unfortunately, the applicability of DTC salts as chemo-protectants is limited by the acute toxicity of free DTC in the body, which biodegrade to different toxic metabolites, such as carbon disulfide, thiourea, and alkyl amines [21]. Nevertheless, when the dithiocarbamates are coordinated to a metal center, the resulting complex is expected to be stable because of the “chelate effect”. This effect makes the complex decomposition unlikely to occur, excluding the possibility of unwanted collateral reactions before reaching the cell. Therefore, our complexes have been designed with the purpose of combining the anticancer activity of the metal center with the protecting action of the sulfur chelating ligand [22].

**Figure 1:**
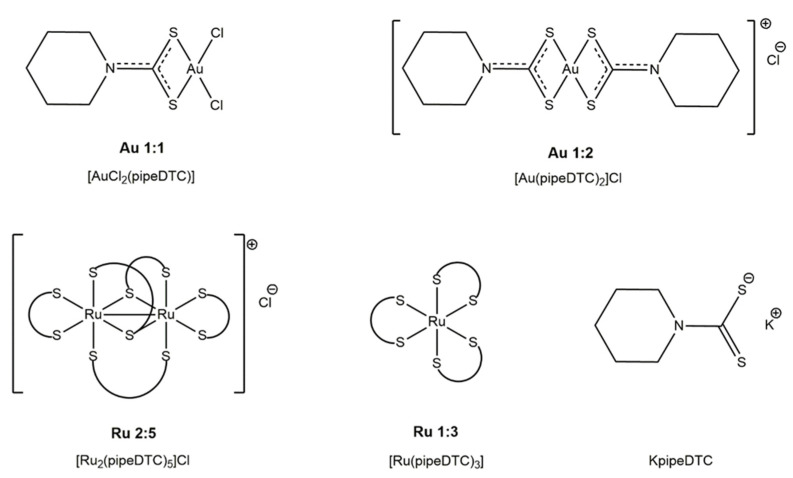
Chemical structures of the ligand K pipeDTC and the gold and ruthenium piperidine dithiocarbamato derivatives. The compounds’ names and their abbreviations are reported here.

In this work, we have focused our attention on two gold(III) and two ruthenium(III) complexes with piperidine dithiocarbamate as the ligand, which show a satisfactory rate of synthesis, purity and stability (Figure 1). We have synthesized the two gold derivatives, although [AuCl_2_(pipeDTC)] has already been reported in the literature with a very different synthetic way from our procedure, in order to compare the behavior of gold complexes with that of ruthenium with the same ligand [23].

After the chemical characterization of the four compounds, special attention has been addressed to the biological evaluation of their antiproliferative activity and their mechanisms of action.

## 2. Results and Discussion

### 2.1. Syntheses and Characterization

The four synthesized compounds were prepared, starting from the common ligand piperidine dithiocarbamato, K pipeDTC. The synthesis of the piperidine dithiocarbamato salt follows the general route, where the acid–base equilibrium, derived from nucleophilic attack of the amine to the electrophilic carbon of CS_2_, is shifted to products by the addition of one equivalent of base.

For the syntheses of the gold piperidine dithiocarbamates, we started by preparing the dinuclear precursor [Au^(I)^(pipeDTC)]_2_ by the reduction in H[AuCl_4_]∙xH_2_O with one equivalent of Na_2_SO_3_, followed by the addition of one equivalent of K pipeDTC salt. Then, the compound [AuCl_2_(pipeDTC)] was synthesized by the oxidative addition of Cl_2_ to a suspension of the previously obtained Au(I)-pipeDTC precursor, whereas [Au(pipeDTC)_2_]Cl was obtained by adding one equivalent of K pipeDTC to a solution of the neutral [AuCl_2_(pipeDTC)] compound.

Concerning ruthenium complexes, both [Ru(pipeDTC)_3_] and [Ru_2_(pipeDTC)_5_]Cl were obtained from the reaction between the precursor RuCl_3_ and the three equivalents of the dithiocarbamato salt K pipeDTC, followed by purification by column gravity chromatography. This final step was crucial to separate the mononuclear paramagnetic [Ru(pipeDTC)_3_] from the dinuclear diamagnetic [Ru_2_(pipeDTC)_5_]Cl.

The starting ligand and the four obtained complexes were characterized by means of several techniques. The proposed stoichiometry of the four compounds was confirmed by elemental analyses, indicating a good correlation between the calculated and found values.

FT-IR spectroscopy was useful to identify the synthesized complexes coordination mode. The presence of a single band *v*(CSS) in the region 950–1050 cm^−1^ indicates a symmetric bidentate coordination mode of the ligand, accordingly to the literature data [24]. Moreover, passing from the pipeDTC ligand to the complexes, a shift in the *v*(N-CSS) and *v*(CSS) to lower wavenumbers is observed, due to the electron-withdrawing character of the metal center, which is mainly evident for gold complexes (see Table 1 for the IR vibrations list).

The ^1^H-NMR spectra were recorded in deuterated acetonitrile CD_3_CN (400.13 MHz, 298 K) to assess the completeness of the dithiocarbamato salt formation. On passing from the free amine to the corresponding DTC ligand, the signal ascribed to the N–H piperidine proton disappears and the C–H signals closest to the nitrogen atom (α protons) shift to lower fields (from 2.68 to 4.34 ppm). Indeed, the electron-withdrawing character of the NCSS moiety of pipeDTC influences the resonance of the α protons, making them resonate at a lower field. On the other hand, the pipeDTC β and γ protons are less affected by the deshielding effect of the nitrogen atom, thus resonating at higher fields compared to the α protons (Table 2). The ^1^H-NMR spectra are influenced by the diamagnetic or paramagnetic character of the metal center. In fact, the spectra of the gold complexes present peaks in the normal range 0–12 ppm, indicating the diamagnetic character of the Au(III) center. On the contrary, the spectrum of the [Ru(pipeDTC)_3_] complex shows a shift in the α protons to lower fields, due to the paramagnetic character of the Ru(III) metal center (Figure S1). On the other hand, the dinuclear complex [Ru_2_(pipeDTC)_5_]Cl spectrum does not present any signal outside the 0–12 ppm range, pointing out the diamagnetic nature of this derivative (Table 2). This is ascribable to the presence of antiferromagnetic coupling of the two Ru(III) ions.

As reported in Figure S2, the complexity of the [Ru_2_(pipeDTC)_5_]Cl spectrum is caused by the different coordination mode adopted by the ligands [25], which led them to be non-magnetically equivalent.

The interaction of Au 1:1, Au 1:2 and Ru 2:5 (see Figure 1 for the structures) in in vitro-active complexes with some model molecules were carried out by ^1^H-NMR spectroscopy. The biologically relevant biomolecules, such as the sulfur-containing amino acids cysteine and methionine, and the histidine, were mimicked by 1-propanethiol (PrSH), dimethyl sulfide (DMS) and 1-methylimidazole (1-MeIm), respectively. The analyses were carried out over 24 h at 310 K, in acetonitrile, as it is the most suitable solvent in terms of volatility and solubility of both the model molecules and the complexes. In fact, the [AuCl_2_(pipeDTC)] complex was poorly soluble in DMSO, while chloroform was not chosen because its volatility did not allow analyses at 37 °C. The molar ratio between the complex and the model molecule was 1:1 for DMS, and 1:2 for PrSH and 1-MeIm [26]. This study highlighted that all the three compounds are inert toward dimethyl sulfide and 1-methylimidazole, but underwent reduction in the presence of the thiol moiety (Figures S3–S11).

### 2.2. Biological Activity

As a preliminary screening, the in vitro cytotoxicity of the four complexes was investigated toward the adenocarcinoma gastric cell line (AGS) and the human colon carcinoma cell line (HCT116). These cell lines were chosen since colon cancer is one of the most frequent tumors in industrialized countries, and gastric cancer is one of the most aggressive. For instance, the overall survival rate at 5 years for a gastric cancer patient is below 25%, and the median survival is below 12 months [27,28]. First, the inhibition of cell proliferation (IC_50_) in two-dimensionally (2D) cultured cells was evaluated over 48 h and 72 h, by means of the MTT assay.

The results were compared to the reference drug cisplatin. The exposure of both the cell lines to increasing concentrations of Au 1:1, Au 1:2 and Ru 2:5 led to a notable inhibition of cell survival, with IC_50_ values below 1 μM over 48 h, which are much lower than those of cisplatin (12–21 μM, respectively). Similar results were obtained for 72 h of treatment. Conversely, Ru 1:3 does not display interesting cytotoxic activity, with IC_50_ values higher than 70 μM. Concerning the HCT116 cell line, the two gold compounds show strong cytotoxicity after 48 h, but this activity diminishes after 72 h (Table 3).

The screening of anticancer drugs on a platform, using 2D-cultured cell lines, does not have the ability to reproduce all the in vivo conditions of the tumor environment [29,30,31]. Based on this observation, we chose to confirm the cytotoxic properties of the most active compounds using HCT116 cultured in 3D, which reproduces the physiological conditions more closely (cells in aggregates presenting different levels of contact with the surroundings). The effect of the drugs was measured by their impact on the size of the sphere, and more quantitatively by assessing cell survival via the resazurin assay. Interestingly, the two gold DTC derivatives and the ruthenium DTC derivatives showed different behaviors in 3D culture compared to the 2D cultures. Unfortunately, both of the gold(III) DTC derivatives did not show any cytotoxic effect in a 3D cell culture, contrary to what was observed in the 2D model.

This inefficacy in the 3D model may suggest a short-term cytostatic effect for these complexes. In this regard, a previous study carried out on gold DTC derivatives of the type [Au^III^Cl_2_(dmdt)] and [Au^III^Br_2_(esdt)] (dmdt= dimethyldithiocarbamato; esdt= ethylsarcosinedithiocarbamate), by real-time xCELLigence analysis, confirmed that they induce a strong cell growth inhibition after 24 h, but the cells restore viability afterwards (26–90 h) [32]. The precise molecular mechanisms that are responsible for the short-term effect or the lack of activity on cell aggregates are unclear and need further investigation. For instance, a drugs stability inside the cells, or the difficulty of the drugs to penetrate within the cell aggregates may play a role.

**Figure 2:**
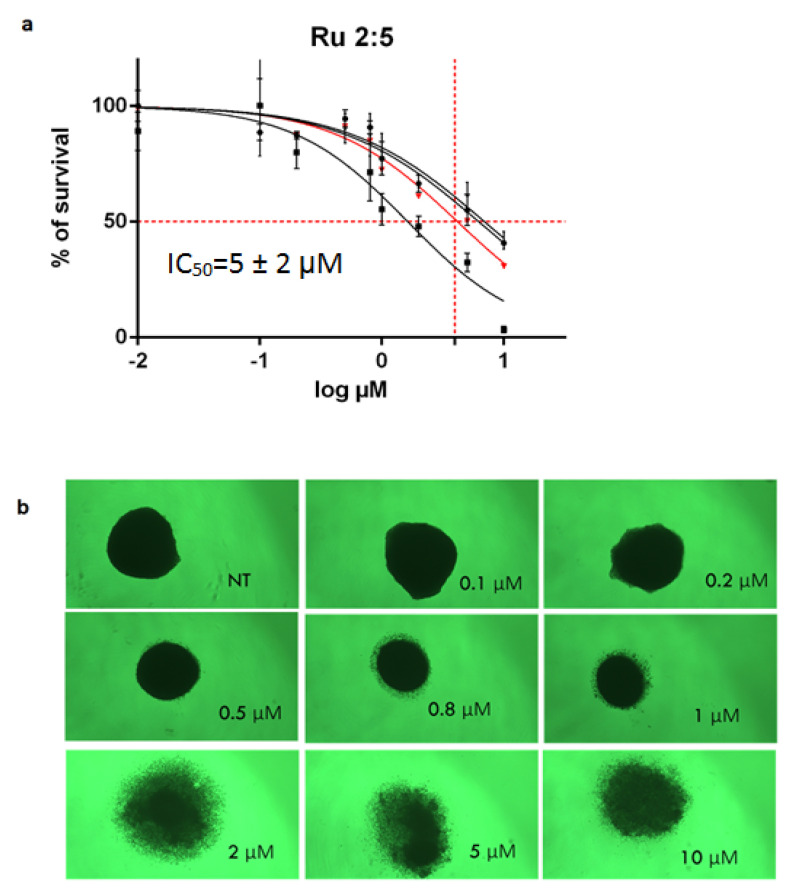
(**a**) IC_50_ values of the 3D-cultured HCT116 cell line. The average of IC_50_ (red line) was calculated from three independent experiments; Cells were cultured for 7 days and then treated for 7 days. (**b**) The 3D spheroids after treatment with different concentrations (0 µM (NT)–10 µM) of [Ru_2_(pipeDTC)_5_]Cl.

On the contrary, [Ru_2_(pipeDTC)_5_]Cl displayed a promising cytotoxic effect in the 3D system (IC_50_ 5 ± 2 μM, Figure 2a), much higher than that of cisplatin (63 ± 25 μM). Moreover, the cytotoxic effect was correlated with the drug concentration-dependent decrease in spheroids’ diameter after the treatment (Figure 2b), thus indicating a progressive decrease in cell–cell interaction, which is associated with cell death.

### 2.3. Investigation of the Mechanism of Action 

**Figure 3:**
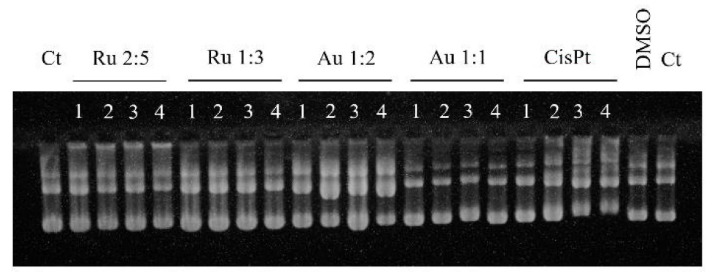
Electrophoretic mobility of pJoJo plasmid DNA in presence of the different DTC derivatives and cisplatin. The plasmid was incubated with the complexes for 24 h at room temperature. For the gold and ruthenium compounds the following concentrations were realized: column 1–4: 15 μM, 30 μM, 45 μM, 60 μM, respectively. For cisplatin: column 1–4: 1 μM, 5 μM, 10 μM, 15 μM, respectively.

DNA has been described to be a target for different types of metal-based complexes, especially ruthenium complexes [33]. In order to understand if DNA was a possible target for the complexes, an EMSA (electrophoretic mobility shift assay) [34] was carried out. This qualitative assay showed that the three active compounds did not interact with DNA as much as cisplatin. In fact, no shift in the bottom band (supercoiled form of plasmid) was observed for the samples incubated with Au 1:1, Au 1:2, and Ru 2:5, when compared with the control (namely, the plasmid without a complex). On the contrary, a shift in the supercoiled plasmid band was observable in the cisplatin-treated samples (Figure 3), as expected, since DNA is its major target [3]. From this preliminary observation, we can conclude that the tested compounds do not interact with DNA when compared to a smaller concentration of CisPt. Anyway, more in-depth studies are needed to confirm this result.

**Figure 4:**
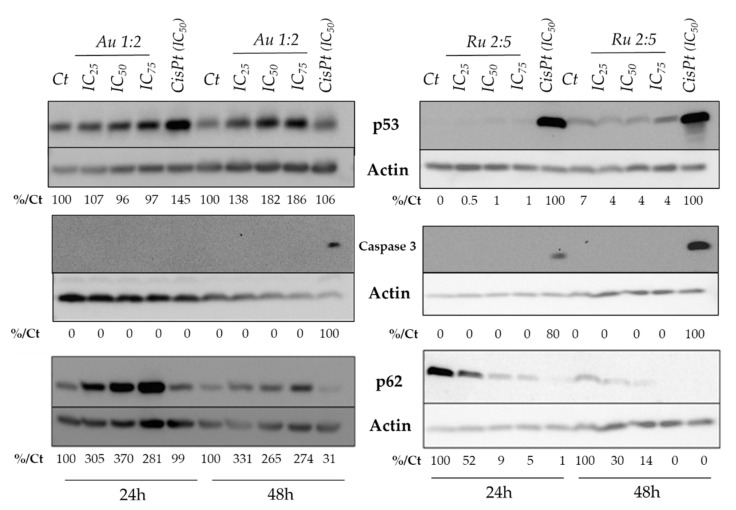
Expression of cleaved caspase-3, p53 and p62. Cisplatin was used as positive control (IC_50_). IC_25_, IC_50_ and IC_75_ were utilized to treat the cells. This image is representative of three independent experiments. The expression of proteins in AGS cells for [Au(pipeDTC)_2_]Cl and in HCT116 cells for [Ru_2_(pipeDTC)_3_] is reported.

It has been elucidated, from previous studies, that ruthenium derivatives are located in various cellular compartments (i.e., ER, mitochondria, nucleus for Ru derivatives, and cytoplasmic area for Au(III) derivatives) [35,36]. In this work, we focused our attention on the possible pathways implicated in causing cell death. To understand the molecular mechanisms involved in the cytotoxicity of the Au 1:2 and Ru 2:5 compounds, we investigated the activation of pro-cell death mechanisms. We first analyzed the protein level of p53, a transcription factor induced by several cellular stresses, which can, in response, induce different types of cell deaths as apoptosis. In addition, it has been demonstrated that over 50% of human tumors are characterized by mutations of p53, leading to the enhanced survivability of tumor cells [37,38,39]. The role of p53 is particularly important in gastric cancer, where the inactivation of p53 correlates with the response to chemotherapy [40]. Western blot was used to measure the p53 protein levels [41]. AGS and HCT116 cells express a wild-type p53. AGS cells were treated over 24 h and 48 h, with both of the two complexes, while HCT116 cells were treated only with the most promising [Ru_2_(pipeDTC)_5_]Cl species, always using cisplatin as a reference drug. The protein levels for p53 were quantified and standardized with the protein levels for actin, to be indicated as a percentage relative to the positive control cisplatin. As expected, cisplatin significantly induced p53 protein levels at 24 h. Different results were obtained for the Au 1:2 and Ru2:5 compounds, as follows: (i) The AGS cells treated with [Au(pipeDTC)_2_]Cl showed a dose-dependent increase in p53 protein levels, starting after 24 h and lasting up to 48 h (Figure 4); (ii) Ru 2:5 did not induce p53 protein levels in both of the cell lines (Figure 4). The fact that this compound did not bind DNA and did not induce p53 expression suggests that this complex may act independently of p53, in contrast to some ruthenium complexes previously described [42], and may resemble more in their mode of action to ruthenium complexes such as RDC11 [43]. This observation is very encouraging, since the ability of compounds that require p53 to induce cell death is rather debilitated in cancer cells with the p53 mutation, which occurs in about 50% of cases [44]. Therefore, antitumor agents triggering cell death independently of p53 pathways could be a promising strategy in the treatment of p53-mutated tumors [42,45].

We then evaluated the cleavage of caspase-3 to estimate if the activity of these compounds is associated with cell apoptosis activated by caspase-3 cleavage [46]. This protein belongs to the family of cysteine proteases, largely known for their role in controlling cell death and inflammation. Caspase-3 is the most widely studied among the effector caspases, since it plays a key role in both the extrinsic and intrinsic pathway of apoptosis [46,47]. For both of the tested compounds in both cell lines, no cleavage of caspase-3 was observed under our conditions, in contrast to the treatment with cisplatin (Figure 4 and Figure S12). This suggested that these complexes probably induce cell death via other mechanisms. Hence, we focused our attention on the possible activation of autophagy. Autophagy is a cellular degradation pathway for the clearance of damaged or superfluous proteins and organelles, which maintain cell homeostasis and viability [48,49]. In this scenario, p62 is a stress-inducible cellular protein that plays a key role as a selective autophagy marker. This protein has a pivotal role, as it is of the main importance in promoting viability, but, on the other hand, a higher expression of p62 is also associated with cell death induction. This last effect in the case of metabolic stress, hypoxia, nutrition deficiency or therapeutic treatment, leads to a non-apoptotic cell death (autophagic cell death, ACD) [49]. Hence, we examined the levels of the p62 protein in cells treated with our complexes. Intriguingly, Au 1:2 induces a dose-response increase in p62 levels, while an opposite behavior is observed in the Ru 2:5-treated cells, which display a dose-dependent decrease in p62 protein levels (Figure 4).

**Figure 5:**
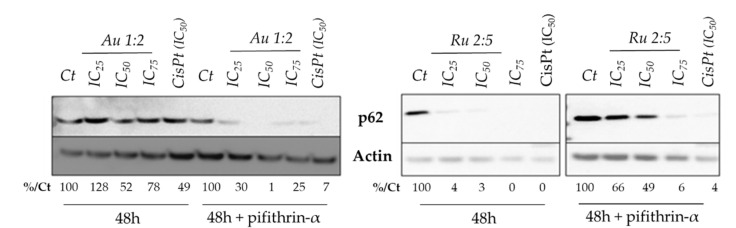
Expression of the autophagy biomarker p62 in HCT116 cells with and without co-treatment with pifithrin-α over 48 h. Cisplatin was used as positive control (IC_50_). This image is representative of three independent experiments.

As Au 1:2 induced p53 protein levels, we investigated whether p53 was playing a role in the p62 increase. To do so, the cells were co-treated over 48 h with pifithrin-α [50], which is a known inhibitor of p53 function [51]. Interestingly, in the presence of the p53 inhibitor pifithrin-α, the complex Au 1:2 decreased the expression levels of p62, whereas pifithrin did not change the activity of the ruthenium derivative (Figure 5). From the collected results, we can propose that the Au 1:2 mechanism of action might be p53-dependent, and that it could act by inducing an enforced autophagy that leads to cell death via a non-apoptotic pathway. Differently, the Ru 2:5 complex seems to not involve autophagy in the cell death pathway, and intriguingly the p53-independent mechanism of action of this compound is also confirmed by the presence of the p53 inhibitor agent.

**Figure 6:**
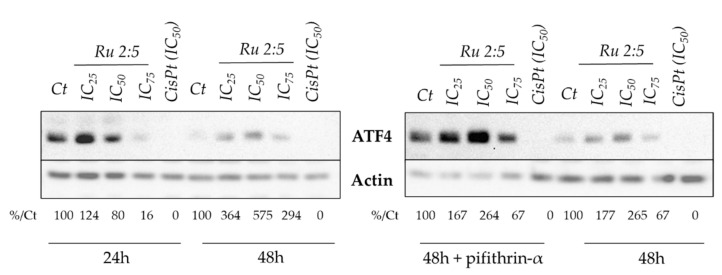
Expression of the ER stress biomarker ATF4 after treatment with Ru 2:5 in HCT116 cell lines. Cisplatin was used as positive control (IC_50_). This image is representative of three independent experiments.

As the ruthenium-based complex has sidestepped all the planned “check points”, in terms of unravelling its mechanism of action, the endoplasmic reticulum (ER)–stress pathway was then considered. Indeed, we previously showed that several ruthenium complexes can induce several markers of the ER stress pathway [35,43,52,53]. Alterations in the folding capacity of the ER, caused by a variety of endogenous and exogenous stimuli, prompt a cellular stress condition known as ER stress. In turn, ER stress activates an intracellular signal transduction pathway, called the unfolded protein response (UPR), which is essential to re-establish ER homeostasis [54]. One of the key regulators of the UPR is the activating transcription factor 4 (ATF4), which monitors the transcription of the essential genes for adaptive functions. Intriguingly, ATF4 can be involved in both a pro-survival and pro-apoptotic role during ER stress conditions, depending on the duration as well as the severity of the ER stress [55]. In fact, under ER stress conditions, an increased translation of selected mRNAs occurs, including ATF4, which monitors the transcription of essential genes for adaptive functions. Focusing on cancer cells, increased levels of ATF4 compared to healthy cells are usually observed, leading to stress adaptation and, consequently, to cancer cell survival. Indeed, the inhibition of ATF4, or a decrease in its levels, results in increased stress, causing cell death [56]. Remarkably, in other cases, the long-term activation of the UPR axis may evoke a paradoxical response via the initiation of apoptotic cell death, highlighting the dual role of this protein in the fate of cancerous cells [55]. Based on this information, we investigated whether our complexes might regulate the protein level of ATF4, as a marker of the induction of the ER stress pathway. The ruthenium complex was able to significantly induce the protein level of ATF4 in cancer cells, in contrast to cisplatin (Figure 6). Interestingly, the response was dose- and time-dependent. At 24 h of treatment, induction was present for a low concentration of the drug, while a high concentration reduced the ATF4 protein level. However, at 48 h of treatment, all of the concentrations were able to induce the ATF4 protein levels. Interestingly, the inhibition of p53 by pifithrin significantly increased the ATF4 protein level, but did not significantly affect the ability of Ru 2:5 to induce ATF4 levels. Hence, this result indicated that the Ru 2:5 complex can induce the expression of at least one marker of the ER stress pathway, suggesting that part of its mode of action may trigger this pathway independently of p53. Additional functional studies will be required to further establish the role of the ER stress pathways in Ru 2:5 cytotoxicity.

## 3. Materials and Methods 

Chemicals and biological materials. The following chemicals were purchased and used as provided by suppliers: tetrachloroauric acid trihydrate (99%), ruthenium(III) chloride trihydrate (≥99%), potassium tetrachloroplatinate(II) (≥99%), manganese dioxide (99%), silver nitrate (≥99%), potassium iodide (99%), sodium sulfite (≥98%), sodium chloride (99%), piperidine (99%), hydrogen chloride (37%), carbon disulfide anhydrous (≥99%) (Sigma-Aldrich). Potassium hydroxide (99%) (VWR). Solvents: dichloromethane, diethyl ether, chloroform, ethanol, ammonium hydroxide (33%) (Sigma-Aldrich). The ^1^H-NMR chemical shifts (δ) of the signals are given in ppm and referenced to residual protons in deuterated solvent acetonitrile-d (CD_3_CN 1.94 ppm) (VWR). DMSO (>99.9%, for biological treatments), methanol (99.8%), Tris-glycine buffer 10x, Dulbecco modified Eagle’s medium (D-MEM), RPMI medium, L-glutamine, penicillin, streptomycin, fetal bovine serum (FBS), trypsin (0.05%, EDTA 0.02% in PBS), in vitro toxicology assay kit (MTT- and resazurin-based), dithiothreitol (DTT) (≥99.0%), Laemmli buffer, sodium dodecyl sulfate (SDS) (≥99.0%), Luminata Forte substrate, Luminata Crescendo substrate, polyacrylamide solution 50 wt.% in H_2_O, N,N,N′,N′-tetramethylethylenediamine (Sigma-Aldrich). Ammonium persulfate (APS) (90%), nitrocellulose membranes, Ready Gel^®^ precast polyacrylamide gels, TEMED, glycerol (BIO RAD). AGS cells, HCT116 cells (American Type Culture Collection, ATCC); pJoJo plasmid was provided by P.Collombat (Université Côte d’Azur, Inserm). Antibodies (Table 1).

*Synthesis of the K PipeDTC ligand*. KOH (2.272 g, 40 mmol) was dissolved in 100 mL of ethanol and 4 mL (40.5 mmol) of piperidine was added with the solution mixture set at 0 °C. Successively, an excess of carbon disulfide (CS_2_) was added, leading to a yellow mixture, which was kept under vigorous stirring for 1 h. Afterwards, the solvent volume was reduced and the product precipitated by adding cold diethyl ether, filtered and washed with cold ethanol, before being dried under vacuum in the presence of P_2_O_5_. Piperidine dithiocarbamate potassium salt: aspect: white solid. Yield: 95%. Anal. Calc. C_6_H_10_KNS_2_ (MW = 199.38 g/mol): C, 36.14; H, 5.06; N, 7.03; S, 32.16. Found: C, 35.84; H, 5.02; N, 7.05; S, 32.22. ^1^H-NMR (CD_3_CN, 300.13 MHz): δ (ppm): 1.50 (m, 4H); 1.60 (m, 2H); 4.34 (t, 4H). Medium FT-IR (KBr): (cm^−1^) = 1417.12 (v N-CSS), 965.00 (v_a_ CSS).

*Synthesis of the precursor [Au^I^(pipeDTC)]_2_*. H[AuCl_4_]∙xH_2_O (714.7 mg, 1.78 mmol) was dissolved in a saturated saline solution at 0 °C, followed by reduction Au(III)→Au(I) with 1 equivalent of Na_2_SO_3_ under stirring. The bright yellow solution turns colorless, and immediately 355.1 mg (1.78 mmol) of K PipeDTC was added, leading to the precipitation of an orange solid. The mixture was left under vigorous stirring for 5 min, and then the precipitate was centrifuged, washed three times with 5 mL of water and dried under vacuum in the presence of P_2_O_5_. Bis(piperidine dithiocarbamate)digold(I): aspect: green–yellow solid. Yield: 98%. Anal. Calc. C_12_H_20_Au_2_N_2_S_4_ (MW = 714.49 g/mol): C, 20.17; H, 2.82; N, 3.92; S, 17.95. Found: C, 20.20; H, 2.82; N, 3.99; S, 17.89. Medium FT-IR (KBr): (cm^−1^) = 1427.69 (v N-CSS), 967.97 (v_a_ CSS).

*Synthesis of [AuCl_2_(pipeDTC)]*. [Au^I^(pipeDTC)]_2_] (0.5 mmol) was suspended in 50 mL of dichloromethane and the mixture was refluxed under stirring. After 10 min, an excess of Cl_2_ (generated in a separate flask by mixing 300 mg of MnO_2_ with 5 mL of concentrated HCl) was gurgled in the Au(I) suspension for 1 h. It was possible to observe that, after some minutes, the solution turned brown with the dissolution of the Au(I) precursor, and then became yellow. Then, the solution was cooled to room temperature and the solvent half-reduced. The compound was precipitated with diethyl ether, washed with 2 × 4 mL of diethyl ether and 2 × 5 mL of deionized water, and the orange product was dried under vacuum in the presence of P_2_O_5_. Dichloro(piperidine dithiocarbamate)gold(III): aspect: dark-yellow solid. Yield: 88%. Anal. Calc. C_6_H_10_AuCl_2_NS_2_ (MW = 428.15 g/mol): C, 16.83; H, 2.35; N, 3.27; S, 14.98. Found: C, 17.02; H, 2.40; N, 3.14; S, 15.02. ^1^H-NMR (CD_3_CN, 400.13 MHz): δ (ppm): 1.78 (s, 6H); 3.75 (s, 4H). Medium FT-IR (KBr): (cm^−1^) = 1582 (v N-CSS), 1006 (v_a_ CSS).

*Synthesis of [Au(pipeDTC)_2_]Cl*. [Au^III^Cl_2_(pipeDTC)](123.1 mg, 0.288 mmol) was dissolved in CHCl_3_ (60 mL), and, in a separate flask, the ligand K pipeDTC (57.3 mg, 0.288 mmol) was dissolved in ethanol (1 mL). The ethanolic solution of the ligand was added dropwise to the Au(III) precursor, and the solution turned from yellow to light orange. After 10 min, the solvent was removed under reduced pressure. The residual solid was taken up with CHCl_3_, and KCl was removed by filtration. The final product was collected by precipitation with n-pentane and dried under vacuum in the presence of P_2_O_5_. Bis(piperidine dithiocarbamate)gold(III) chloride: aspect: yellow–orange solid. Yield: 82%. Anal. Calc. C_12_H_20_AuN_2_S_4_Cl (MW = 552.98 g/mol): C, 26.04; H, 3.61; N, 5.06; S, 23.19. Found: C, 25.86; H, 3.93; N, 4.83; S, 23,32. ^1^H-NMR (CD_3_CN, 400.13 MHz): δ (ppm): 1.79 (m12H); 3.80 (m, 8H). Medium FT-IR (KBr): (cm^−1^) = 1560 (v N-CSS), 1003 ( v_a_ CSS).

*Syntheses of [Ru_2_(pipeDTC)_5_]Cl and [Ru(pipeDTC)_3_]*. Ruthenium(III)chloride trihydrate (818.9 mg, 3.13 mmol) and K pipeDTC (187.3 mg, 9.39 mmol) were separately dissolved in 5 mL of deionized water. The solution of ligand was added to Ru(III), and stirred at room temperature for 1 h, yielding a brown precipitate that was centrifuged, washed with 2 × 10 mL of deionized water and dried under vacuum in the presence of P_2_O_5_. After 24 h, the isolated product was re-dissolved in 20 mL of CH_2_Cl_2_ and the solution filtered to remove Ru oxides by-products. The obtained solution was purified via silica gel chromatography. A gradient from CH_2_Cl_2_ 100% to CH_2_Cl_2_/MeOH 90%:10% was used to first elute the dark-green mononuclear complex [Ru(pipeDTC)_3_] and then the brown dinuclear derivative [Ru_2_(pipeDTC)_5_]Cl as a mixture of α and β isomers. Successively, the mixture of α, β-[Ru_2_(pipeDTC)_5_]Cl was isomerized to the thermodynamically stable β-[Ru_2_(pipeDTC)_5_]Cl by reflux in methanol for 8 h. Both the mononuclear and the β-dinuclear complexes were re-precipitated from CH_2_Cl_2_ diethyl ether, washed with n-pentane, and dried in vacuum in the presence of P_2_O_5_. β-pentakis(piperidine dithiocarbamate)diruthenium(III)chloride: aspect: brown solid. Yield: 30%. Anal. Calc. C30H50Ru2ClN_5_S_10_ (MW = 1039.00 g/mol): C, 34.64; H, 4.81; N, 6.73; S, 30.85. Found: C, 33.01; H, 4.73; N, 6.50; S, 30.37. ^1^H-NMR (CD_3_CN, 400.13 MHz): δ (ppm): 1.75 (m, 10H); 3.88 (m, 6H). Medium FT-IR (KBr): (cm^−1^) = 1505, 1441 (v N-CSS), 1001 (v_a_ CSS). Tris(piperidine dirhiocarbamate)ruthenium(III): aspect: dark-green solid. Yield: 34%. Anal. Calc. C_18_H_30_RuN_3_S_6_ (MW = 581.91 g/mol): C, 37.13; H, 5.16; N,7.22; S, 33.07. Found: C, 37.19; H, 5.01; N, 7.00; S, 33.97.**^1^**H-NMR (CD_3_CN, 400.13 MHz): δ (ppm): 26.02 (m, 6H); 21.95 (m, 6H); 1.01 (m, 12H); 3.95 (m, 6H). Medium FT-IR (KBr): (cm^−1^) 1488, 1455, 1440 (v N-CSS), 1002 (v_a_ CSS).

### 3.1. Cell Lines and Culture Conditions

The 2D cell culture. Gastric adenocarcinoma AGS cells were maintained in RPMI medium, while the colorectal cancer cells HCT116 were maintained in DMEM high-glucose medium, both with 10% of FBS and incubated in presence of 5% CO_2_ at 37 °C.

To transfer cells from the Petri cell plate to the test plates (polystyrene, 96-well), the following procedure was used: After removal of the medium, cells were washed with 5 mL of EDTA and thus 5 mL of trypsin 0.5% was added. The Petri cell plate was then incubated for 3–5 min to detach cells from the plate surface. After that, 10 mL of culture medium was added to stop the trypsin reaction and then the suspension was transferred to a falcon and centrifuged for 5 min at 800 rpm, to collect the cells on the bottom. Subsequently, the supernatant was carefully removed, and 20 mL of new medium was added. The cell suspension was diluted at a final concentration of 10^5^ cells/mL and 100 μL of the cellular suspension was seeded in 96-well microplates and incubated at 37 °C in a 5% CO_2_ atmosphere until reaching 70/80% confluence. The day after seeding, cells were treated with vehicle (control; namely, DMSO or saline solution) or each compound (dissolved in the vehicle DMSO/saline solution, 0.8% *v*/*v*) in fresh culture medium at the defined concentrations (i.e., 10 μM, 5 μM, 2 μM, 1 μM, 0.5 μM and in some cases lower) for 48 or 72 h. Cell viability was evaluated via MTT assay, according to standard procedures [57,58].

Cells were treated for 48 h and 72 h and then the medium was completely removed and replaced with 100 μL of a 3-(4,5-dimethylthiazol-2-yl)-2,5-diphenyltetrazolium bromide (10% MTT solution in PBS, phosphate-buffered saline solution, pH 7.4), and incubated for 2 h at 37 °C. After removing incubation medium, formazan crystals were dissolved in 100 μL solution of DMSO, and MTT reduction was quantified by measuring the absorbance at 570 nm. The percentage of surviving cells was calculated from the ratio of absorbance between treated and untreated cells. Each treatment was performed in triplicate.

Cytotoxicity data were expressed as IC_50_ values, i.e., the concentration of the test complex inducing 50% reduction in cell numbers compared with control cultures.

Cisplatin (in saline solution, NaCl 0.9% *w/v*) was used as reference drug.

The 3D cell culture. HCT116 cells were seeded at a density of 300 cells/well in ad hoc conceived 96-well plates. In particular, 50 μL of cell suspension in DMEM (6000 cells/mL) was dispensed in each well. Therefore, cells were incubated in a 5% CO_2_ incubator at 37 °C for seven days and then 50 μL of medium drug solution at different concentrations was added, without removing the previous medium. After that, cells were incubated again for seven days and then incubated overnight with resazurin 10%. The optical density of each well was quantified at 590 nm, using a multi-well plate reader (TriStar2 Berthold).

EMSA. Samples of plasmid DNA pJoJo (kindly provided by Prof. P. Collombat, Université Côte d’Azur, Inserm) were incubated overnight with Ru 2:5, Au 1:2, Au 1:1 in a 20 mM NaCl solution (final volume 20 μL) at room temperature in the dark. The samples were subjected to electrophoresis on 0.7% agarose gels, running at 25 °C with TAE buffer with a voltage set at 50 V. The gels were then stained with EtBr, followed by detection at 302 nm. The following four different concentrations of complexes were used: (i) 15, 30, 45, 60 μM for Au 1:1, Au 1:2 and Ru 2:5, and (ii) 1, 5, 10, 15 μM for cisplatin.

Western Blot. Cells were seeded in 6-well plates at a density of 5∙10^5^ cells/well and grown in a 5% CO_2_ incubator at 37 °C. In order to avoid cell scratching, cells were then treated with a lysis buffer (Laemmli 2x buffer [59]) with the addition of DTT, and the protein suspension was denatured at 100 °C for 5 min. Next, proteins were solved on a SDS-PAGE gel and transferred to BIORAD nitrocellulose membrane. Membranes were then treated with 20% methanol/10% triglycine solution, blocked with 5% milk powder in PBS + 0.05% Tween 20 for 30 min, and subsequently incubated overnight with primary antibody in blocking solution at 4 °C. Following washes with 5% milk PBS, membranes were incubated with 1:5000 HRP-conjugated secondary antibodies for 2 h at room temperature and washed with PBS + 0.05% Tween 20. HRP reactivity was revealed using Luminata Crescendo or Luminata Forte (Syngene). β-actin was used as a loading control because of its ubiquitarian expression across all eukaryotic cell types [60].

In Table 4 all the primary and secondary antibodies used to detect the proteins in this work and their dilutions are reported.

### 3.2. Instrumentation 

Elemental analyses of carbon, nitrogen and hydrogen were carried out at the Microanalysis Laboratory (Chemistry Dept., University of Padova) with a Carlo Erba mod. 1108 CHNS-O microanalyzer.

Near-FT-IR spectra (400–4000 cm^−1^) were registered at room temperature (32 scans, resolution 2 cm^−1^) by Nicolet Nexus 5SXC spectrophotometer. KBr pellets of samples were prepared according to standard procedures. Spectra were processed with OMNIC 5.2 (Nicolet Instrument Corporation). ^1^H-NMR spectra were recorded at 298 K on a Bruker Advance DEX400 spectrophotometer equipped with a BBI-5 mm z-field gradient probe-head, and a Silicon Graphics O_2_ workstation, operating in Fourier transform. Typical acquisition parameters are as follows (^1^H: 400.13 MHz): 128 transients spectral width 7.5 kHz, 2 k data points and a delay time of 1.0 s. Spectra were processed by using sine-square weighting with a resolution of 1.0/3.0 Hz and a line-broadening threshold of 0.3/1.0 Hz. Data processing was carried out by means of MestReNova version 6.2.0 (Mestrelab Research S.L.). Peak multiplicity has been described as follows: s (singlet), t (triplet), q (quartet), sext. (sextet) and m (multiplet). The ^1^H-NMR study of the interaction between selected models of biomolecules and the metal–dithiocarbamato complexes was carried out dissolving the metal–dithiocarbamato derivative (i.e., [AuCl_2_(pipeDTC)], [Au(pipeDTC)_2_]Cl, and β-[Ru_2_(pipeDTC)_5_]Cl) in deuterated acetonitrile CD_3_CN (0.75 mL) to form 18–19 mM solutions. The sample was put into an NMR tube and the biomolecule was added at the right ratio (1:1 complex:model molecule for DMS and 1-MeIm, and 1:2 for PrSH).

Cell cultures were incubated at 37 °C in a 5% CO_2_-controlled atmosphere in Heracell 150i CO_2_ incubator (Thermo Scientific). Cellular viability was determined by absorbance measurements at 570 nm (MTT) and 595 nm (resazurin) using a plate reader TriStar^2^ S LB 942 modular monochromator multimode reader (Berthold Technologies).

Data were processed by GraphPad Prism version 6.01 software.

Cells were controlled daily to check the confluence with the inverse microscope Motic AT31E.

To assess the Western blot the membranes were detected with multi-fluorescence and chemiluminescence imaging system G:BOX mini Pixi4 (Syngene).

The detection of agarose gels was carried out by AlphaImager mini spectrophotometer.

## 4. Conclusions 

Among the various strategies to fight cancer, the one focused on metal-based chemotherapy could be a winning approach. Our twenty years of studies, focused on the design of new drugs with a lower toxicological impact than cisplatin, have highlighted compounds based on gold and ruthenium, with truly interesting antineoplastic characteristics. In this work, the anticancer activity of four gold and ruthenium complexes, with the ligand piperidine dithiocarbamate, was assessed. Two frequent and aggressive types of cancer, human gastric adenocarcinoma (AGS) and colorectal carcinoma (HCT116), were tested in 2D cell cultures, and IC_50_ values lower than or close to 1 μM were found after 24 h or 72 h of treatment, for three of the tested compounds.

To get closer to the tridimensional aspect of a tumor, we subsequently referred to a 3D model of HCT116 cells. These 3D experiments pointed out a very different behavior of the two class of compounds, namely, the gold and the ruthenium derivatives. The results for the gold-based complexes, seen in light of previous published data, suggest a short-term cytostatic effect. The exact causes of this lack of efficacy in 3D cultures are unclear and need further investigation. One explanation might be a reduced ability of the gold compound to penetrate within the cell aggregates.

On the contrary, Ru 2:5 displayed a promising cytotoxic effect of one order of magnitude greater than cisplatin. The interaction NMR experiments of the different complexes with model molecules, mimicking the major biological competitor of the ligands, pointed out their general stability towards histidine and methionine, but also showed that they undergo reduction in the presence of the model molecule mimicking cysteine. This result, together with the fact that the EMSA assay showed that they do not strongly bind DNA as cisplatin does, could be the key to start understanding the real mechanism of action.

While having the same ligands, gold and ruthenium derivatives have been shown to exploit different pathways in preventing cancer cell growth. In particular, Ru 2:5 has been shown to not involve neither the apoptotic pathway nor the autophagic one, displaying a very promising p53-independent mechanism of action. This is a very important feature, since many tumors have p53 as a mutated gene, leading to a decreased efficacy of many drugs, including cisplatin, which we consider as a reference in our comparative studies. The ability of this compound to sidestep the p53 pathway could be of strategic importance in the incessant designing of more effective anticancer drugs. This striking experimental evidence prompted us to deepen the study of this promising [Ru_2_(pipeDTC)_5_]Cl compound, investigating the ER stress pathway. The results pointed out a general downregulation of the ATF4 protein, a biomarker of ER stress with a key role in cell recovery, which in this case could stimulate onco-suppressor action.

Even if the understanding of the type of induced cell death is troublesome, since some regulatory mechanisms are shared between the different types of cell death, the results collected in this work represent a promising starting point for already planned future studies on these complexes with very interesting, but still unclear, mechanisms of action.

## Figures and Tables

**Table 1:** List of the main IR vibrations of the metal complexes.

Compound	*v*(N-CSS) (cm^−1^)	*v*(CSS) (cm^−1^)
K pipeDTC	1417	965
[Au(pipeDTC)_2_]Cl	1560	1003
[AuCl_2_(pipeDTC)]	1582	1006
[Ru(pipeDTC)_3_]	1488, 1455, 1440	1002
[Ru_2_(pipeDTC)_5_]Cl	1505, 1441	1001

**Table 2:** List of the proton chemical shifts (ppm) of the N–H piperidine, and the DTC derivatives.

Compound	α-CH_2_ (ppm)	β-CH_2_ (ppm)	γ-CH_2_ (ppm)
piperidine	2.68	1.40	1.40
K pipeDTC	4.34	1.50	1.60
[AuCl_2_(pipeDTC)]	3.75	1.78	1.78
[Au(pipeDTC)_2_]Cl	3.80	1.79	1.79
[Ru(pipeDTC)_3_]	26.02 21.95	1.01	3.95
[Ru_2_(pipeDTC)_5_]Cl	3.88	1.55	1.55

All the spectra were recorded with a 400.13 MHz spectrometer at 298 K in CD_3_CN.

**Table 3:** IC_50_ values (μM) for the different compounds (Figure 1), calculated after 48 h and 72 h.

	48 h		72 h	
Compound	HCT116	AGS	HCT116	AGS
Au 1:1	0.3 ± 0.2	0.9 ± 0.2	1.1 ± 0.7	0.4 ± 0.2
Au 1:2	0.7 ± 0.5	0.3 ± 0.2	1.0 ± 0.2	0.4 ± 0.2
Ru 2:5	0.28 ± 0.07	0.65 ± 0.08	0.25 ± 0.09	0.5 ± 0.1
cis-Pt	12 ± 2	21 ± 2	5.6 ± 0.6	8 ± 2
Ru 1:3	>70	>70	>70	>70

The error was calculated as standard deviation of the average IC_50_ values using three or more independent experiments.

**Table 4:** Detected proteins, primary and secondary antibodies, relative dilutions and substrates used in this work.

Protein	1° Antibody	Dilution	2° Antibody	Dilution	Substrate
caspase-3	polyclonal rabbit (Cell Signaling)	1:1000	anti-rabbit (goat) (GE HealthCare)	1:5000	Luminata Forte
p62	monoclonal mouse (Santa Cruz)	1:1000	anti-mouse (goat) (GE HealthCare)	1:5000	Luminata Crescendo
p53	polyclonal rabbit (Santa Cruz)	1:1000	anti-rabbit (goat) (GE HealthCare)	1:5000	Luminata Forte
ATF4	Polyclonal rat (BioLegend)	1:500	anti-rat (rabbit) (GE HealthCare)	1:10,000	Luminata Forte
actin-β	Monoclonal mouse (Chemicon)	1:1000	anti-mouse (goat) (GE HealthCare)	1:10,000	Luminata Crescendo

## Data Availability

The ^1^H-NMR spectra presented in this study are available in Supplementary material.

## Supplementary Materials

The following are available online. ^1^H-NMR spectra of the ruthenium compounds (Figures S1 and S2), ^1^H-NMR spectra of the interaction between complexes and model molecules (Figures S3–S11), synthesis of cisplatin.

## Sample Availability

Samples of the compounds are not available from the authors.
