# Supplementary material for: Gold(III) to Ruthenium(III) Metal Exchange in Dithiocarbamato Complexes Tunes Their Biological Mode of Action for Cytotoxicity in Cancer Cells
Source: Molecules. 2021 Jul 3;26(13):4073. doi: 10.3390/molecules26134073 (PMC8271683; doi:10.3390/molecules26134073)
Supplement: Supplementary file 1 [file molecules-26-04073-s001.zip › molecules-1256691-supplementary.pdf]

Supporting Information

Synthesis, characterization and biological activity evaluation of Gold(III)- and Ruthenium(III)-dithiocarbamato complexes with anticancer properties

Maria Dalla Pozza[1], Christophe Orvain[2], Leonardo Brustolin[1], Chiara Nardon[1], Nicolò Pettenuzzo[1], Christian Gaiddon[2]*, Dolores Fregona[1]*

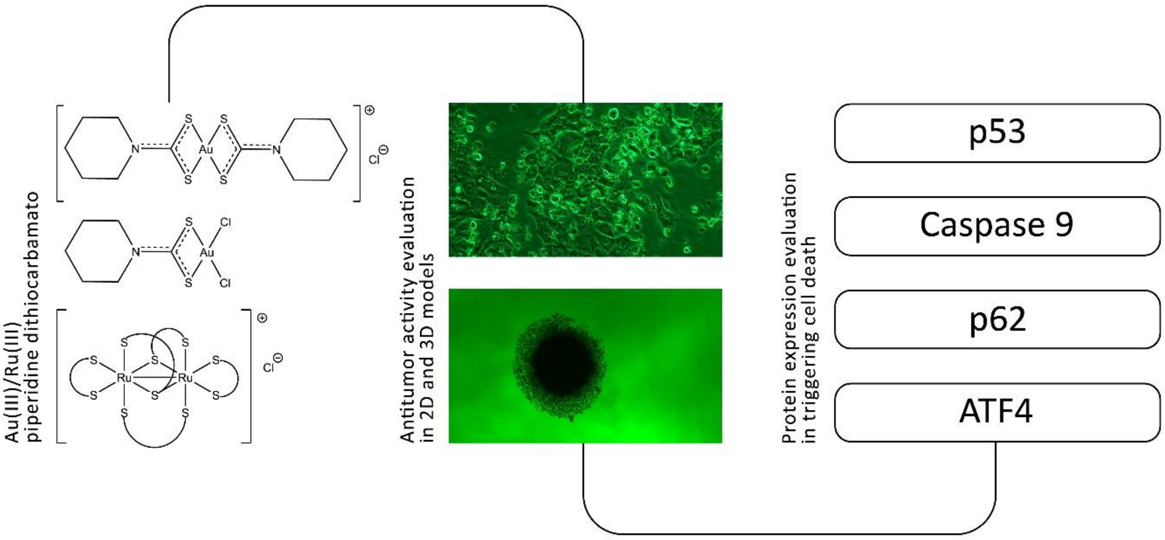

TABLE OF CONTENTS

Figure S1. 1H-NMR spectrum of [Ru(pipeDTC)3] S3

Figure S2. 1H-NMR spectrum of [Ru2(pipeDTC)5]Cl S3

Figure S3. 1H-NMR spectrum of [AuCl2(pipeDTC)] with 1 equivalent of dimethylsulfide S4

Figure S4. 1H-NMR spectrum of [Au(pipeDTC)2]Cl with 1 equivalent of dimethylsulfide. S4

Figure S5. 1H-NMR spectrum of [Ru2(pipeDTC)5]Cl with 1 equivalent of dimethylsulfide. S5

Figure S6. 1H-NMR spectrum of [AuCl2(pipeDTC)] with 2 equivalents of 1-methylimidazole S5

Figure S7. 1H-NMR spectrum of [Au(pipeDTC)2]Cl with 2 equivalents of 1-methylimidazole S6

Figure S8. 1H-NMR spectrum of [Ru2(pipeDTC)5]Cl with 2 equivalents of 1-methylimidazole S6

Figure S9. 1H-NMR spectrum of [AuCl2(pipeDTC)] with 2 equivalents of 1-propanethiol. S7

Figure S10. 1H-NMR spectrum of [Au(pipeDTC)2]Cl with 2 equivalents of 1-propanethiol S7

Figure S11. 1H-NMR spectrum of [Ru2(pipeDTC)5]Cl with 2 equivalents of 1-propanethiol. S8

Synthesis of Cisplatin S8

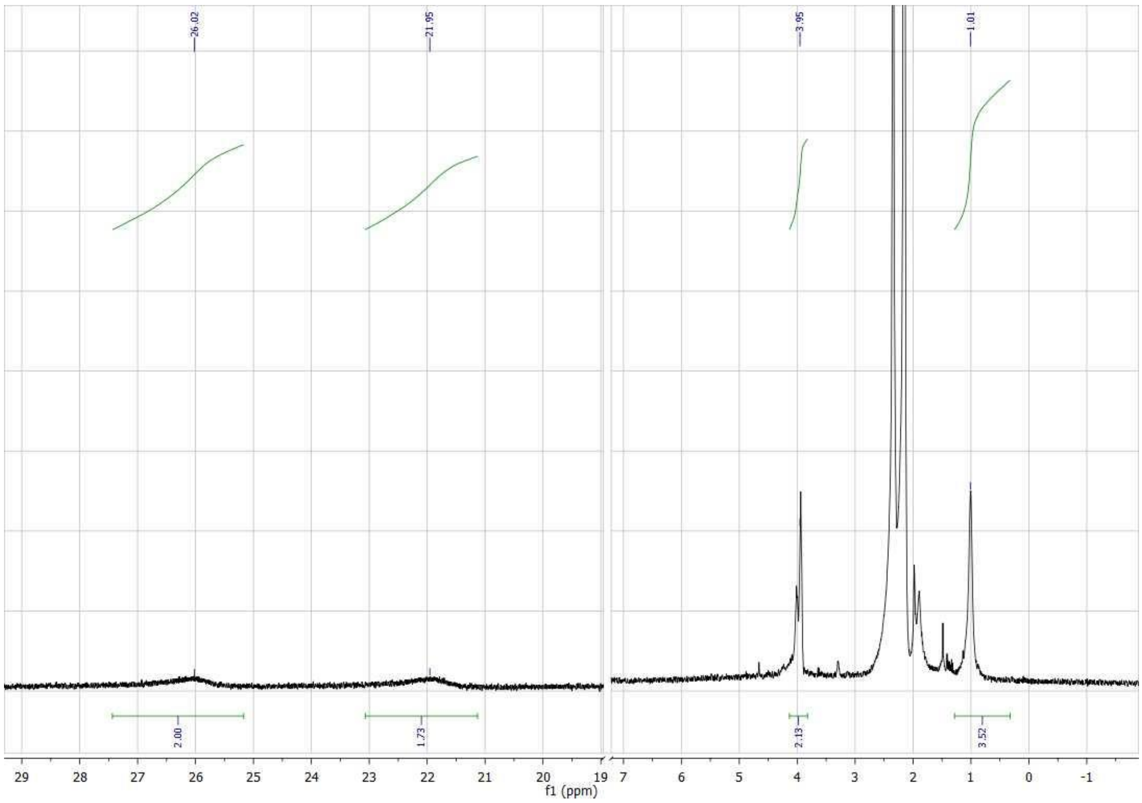

Figure S1: ^{1}H-NMR spectrum of [Ru(pipeDTC)$_3$]

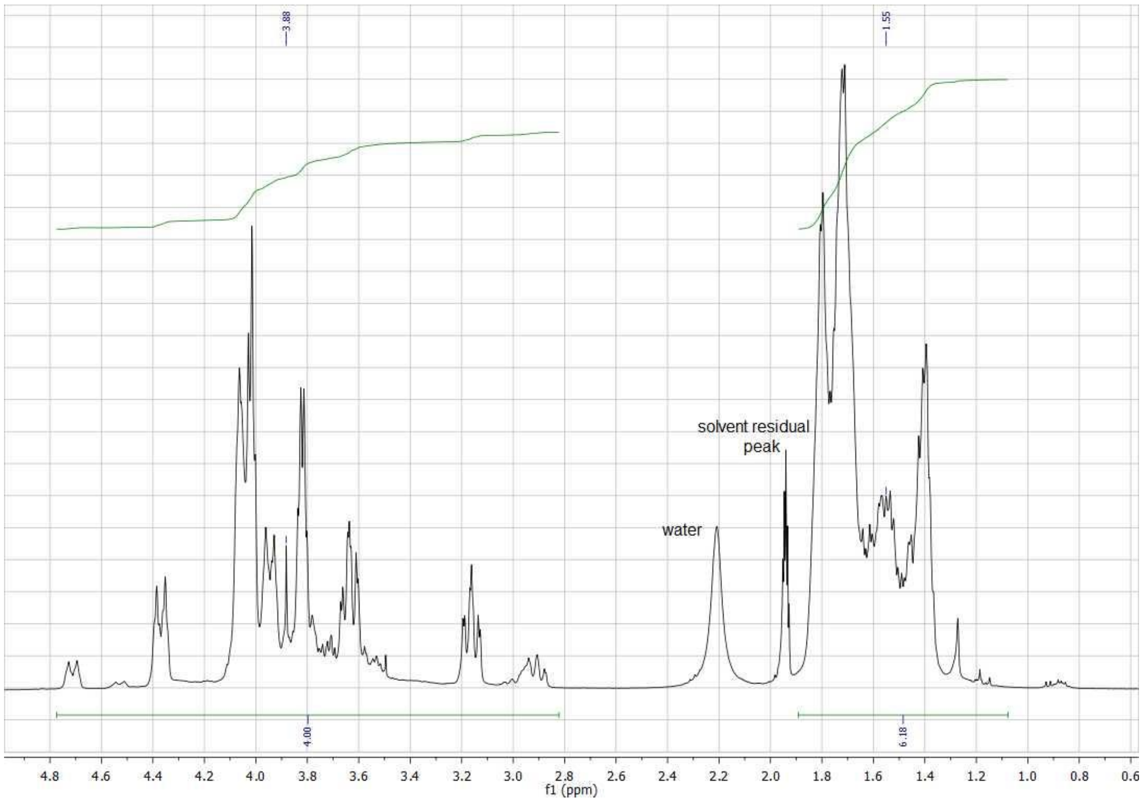

Figure S2: ^{1}H-NMR spectrum of [Ru$_2$(pipeDTC)$_5$]Cl

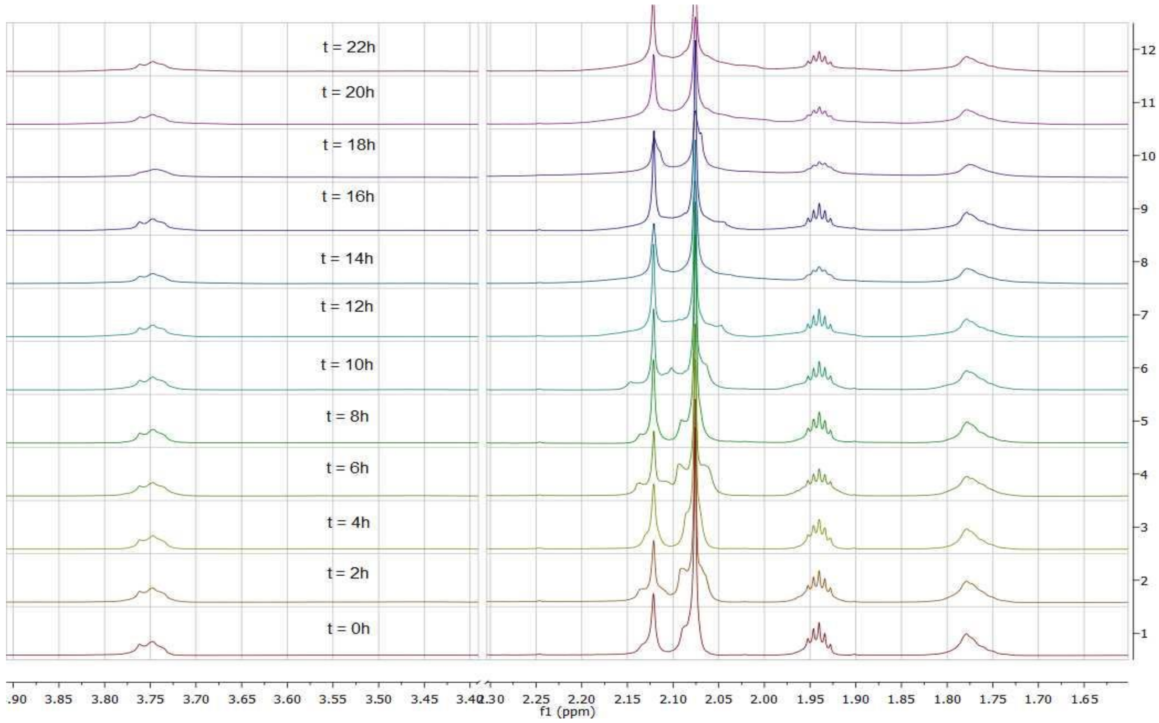

Figure S3: ^{1}H-NMR spectrum of $[AuCl_2(pipeDTC)]$ with the addition of 1 equivalent of dimethylsulfide. The spectrum was recorded at 310 K in CD_3CN.

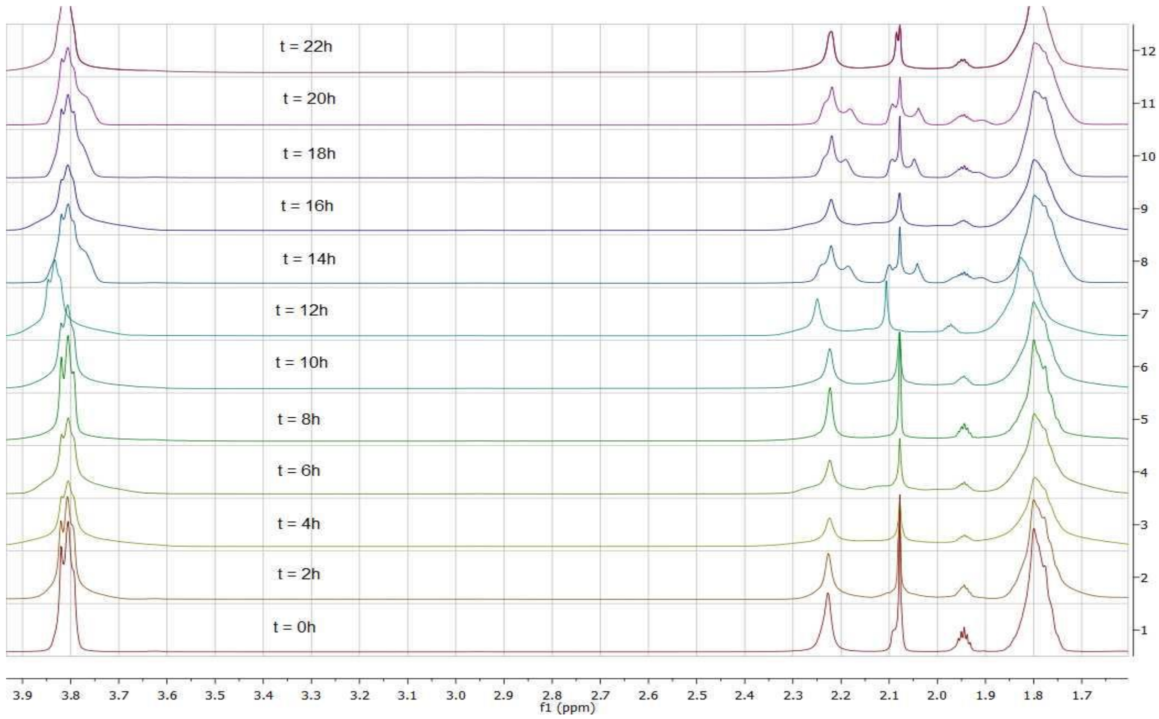

Figure S4: ^{1}H-NMR spectrum of $[Au(pipeDTC)_2]Cl$ with the addition of 1 equivalent of dimethylsulfide. The spectrum was recorded at 310 K in CD_3CN.

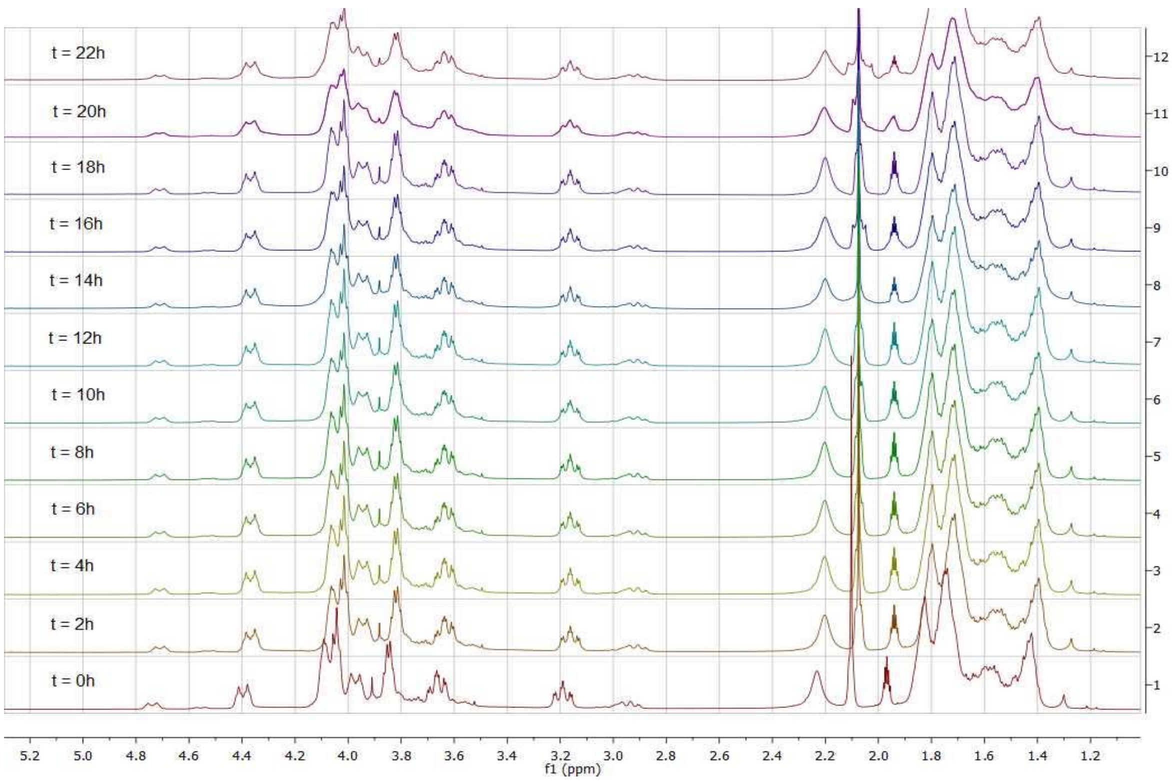

Figure S5: ^{1}H-NMR spectrum of $[Ru_2(pipeDTC)_5]Cl$ with the addition of 1 equivalent of dimethylsulfide. The spectrum was recorded at 310 K in CD_3CN.

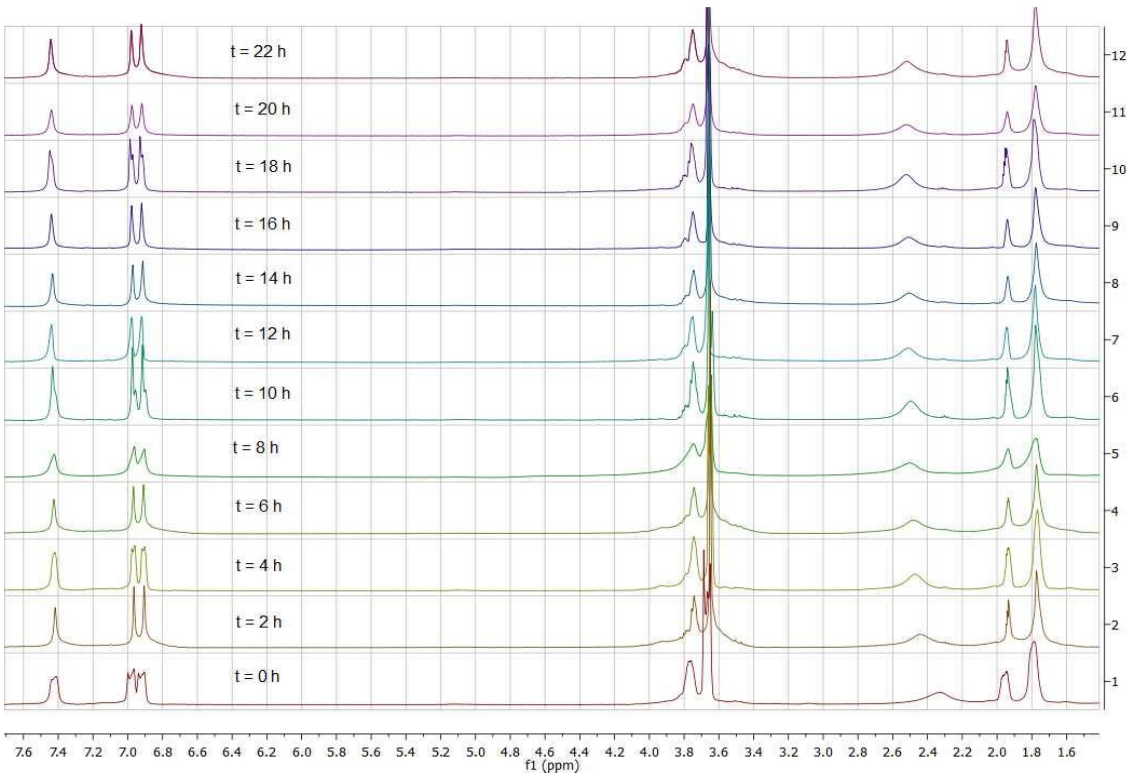

Figure S6: ^{1}H-NMR spectrum of $[AuCl_2(pipeDTC)]$ with the addition of 2 equivalents of 1-methylimidazole. The spectrum was recorded at 310 K in CD_3CN.

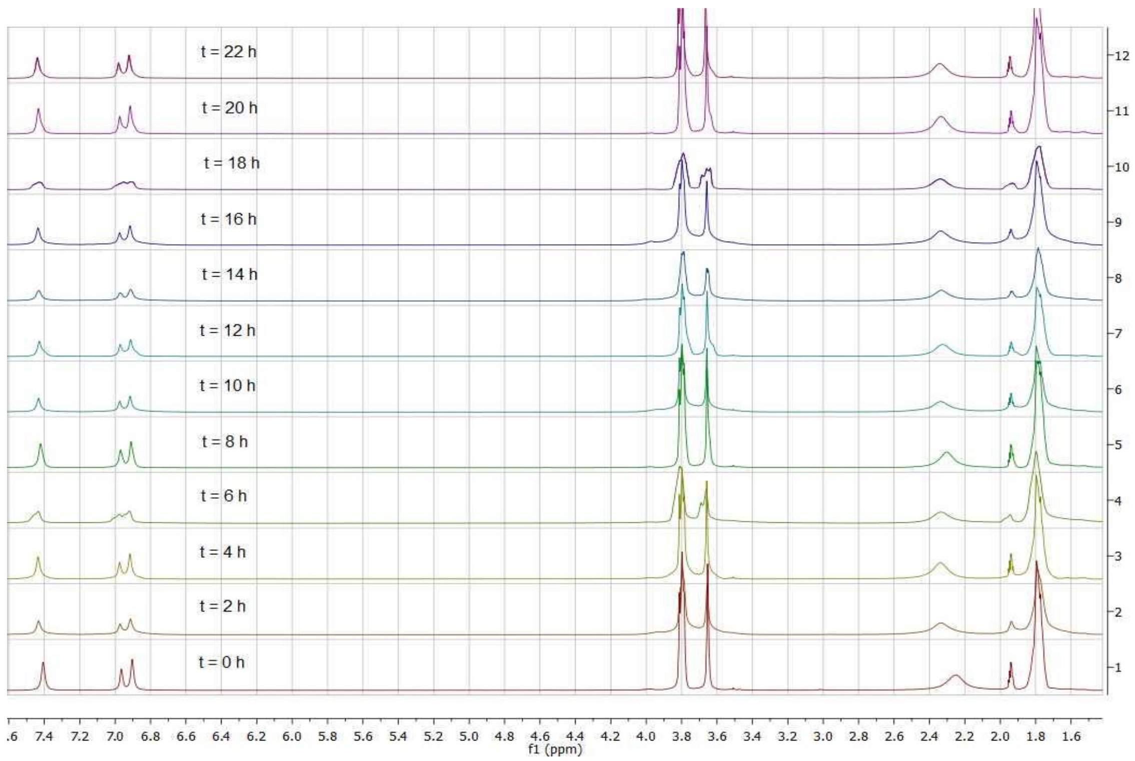

Figure S7: ^{1}H-NMR spectrum of [Au(pipeDTC)$_2$]Cl with the addition of 2 equivalents of 1-methylimidazole. The spectrum was recorded at 310 K in CD_3CN.

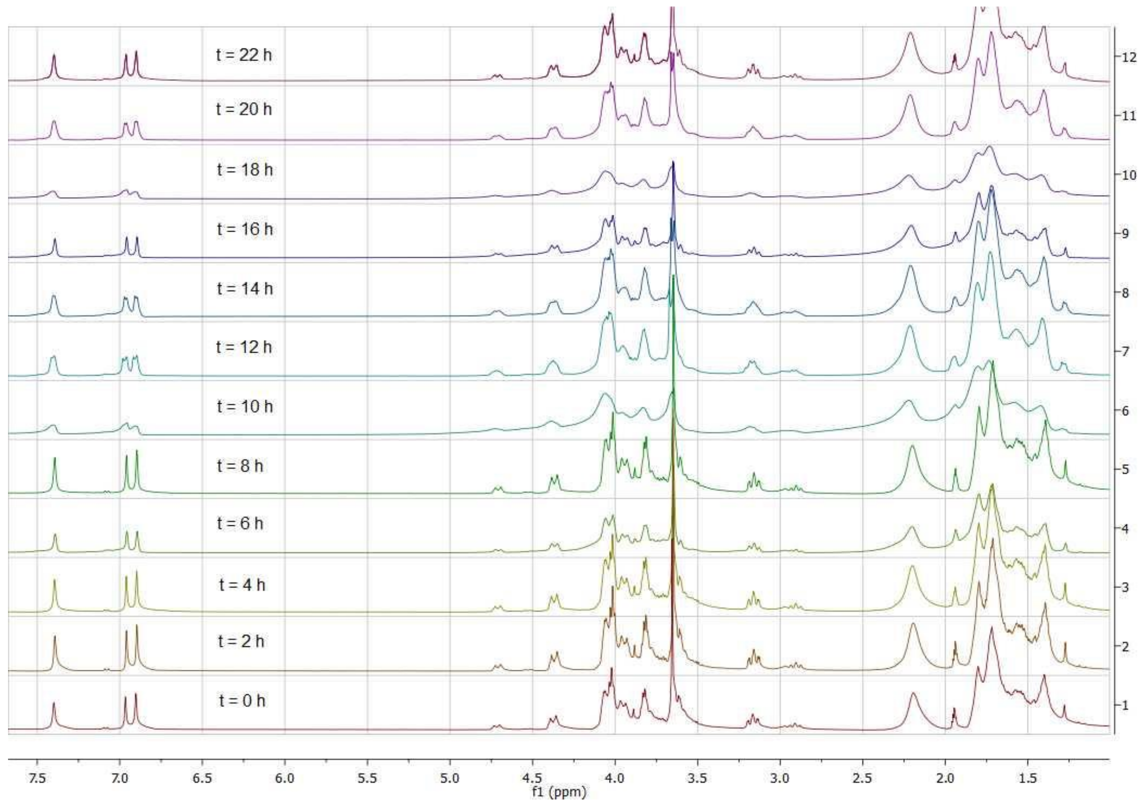

Figure S8: ^{1}H-NMR spectrum of [Ru_2(pipeDTC)$_5$]Cl with the addition of 2 equivalents of 1-methylimidazole. The spectrum was recorded at 310 K in CD_3CN.

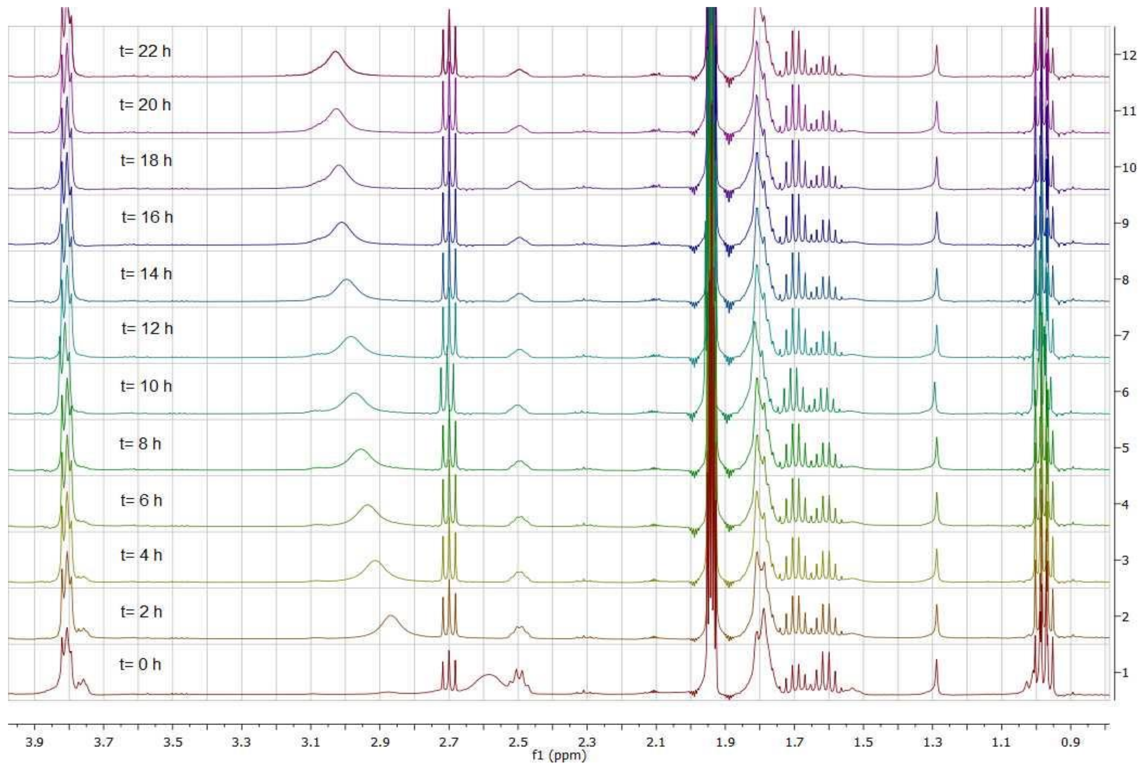

Figure S9: ^{1}H-NMR spectrum of [$AuCl_2$(pipeDTC)] with the addition of 2 equivalents of 1-propanethiol. The spectrum was recorded at 310 K in CD_3CN.

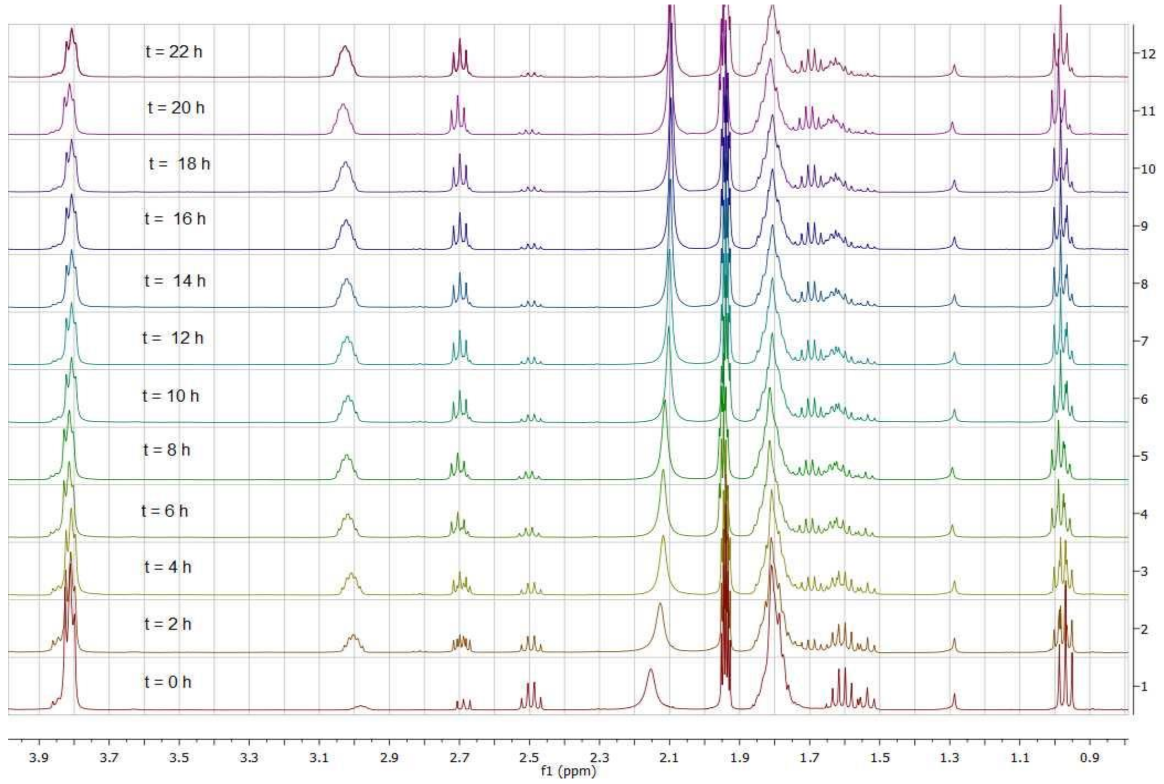

Figure S10: ^{1}H-NMR spectrum of [Au(pipeDTC)$_2$]Cl with the addition of 2 equivalents of 1-propanethiol. The spectrum was recorded at 310 K in CD_3CN.

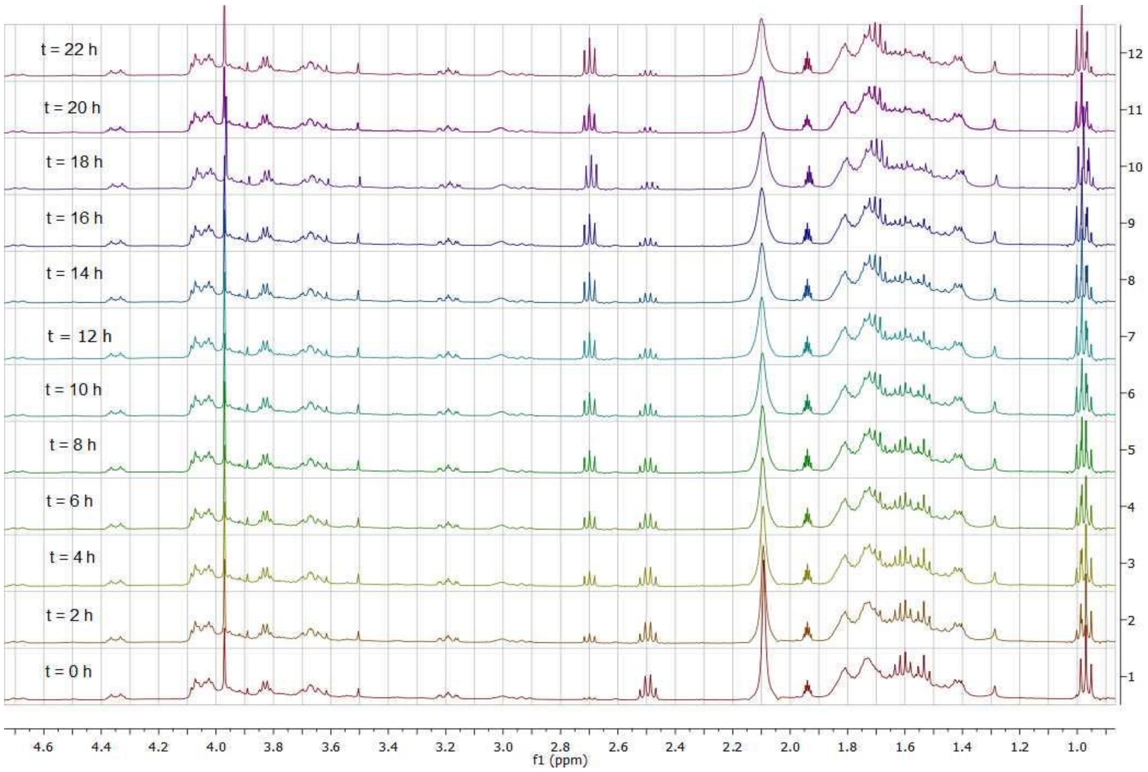

Figure S11: ^{1}H-NMR spectrum of $[Ru_2(pipeDTC)_5]Cl$ with the addition of 2 equivalents of 1-propanethiol. The spectrum was recorded at 310 K in CD_3CN.

Synthesis of Cisplatin

A solution of $K_2[PtCl_4]$ (506.2 mg, 1.22 mmol) in 8 mL of deionized water was mixed with KI (1.635 g, 9.84 mmol) under stirring at room temperature for 30 minutes. Successively, after addition of concentrated NH_4OH (2.44 mmol, 320 µL), the formation of a yellow precipitate of cis-$[PtI_2(NH_3)_2]$ was observed. The solid was centrifuged and washed with 2x 3 mL of cold deionized water and then with 2x 3 mL of cold EtOH and dried under vacuum. After 24 hours, the di-iodo Pt(II) complex (1.03 mmol, 500 mg) was dissolved in deionized water (8 mL) and treated with $AgNO_3$ (2.07 mmol, 351.7 mg). The mixture was stirred at 55°C overnight, then it was filtered to remove AgI and the obtained colourless solution was added of NaCl (4.12 mmol, 244 mg) under stirring. After 30 minutes, a yellow precipitate was formed. The solution was cooled at 4°C and the precipitate was separated, washed with cool water and dried in vacuum in the presence of P_2O_5. Aspect: yellow solid. Yield: 87%. Anal. Calc. Pt $N_2H_6Cl_2$ (MW= 300.05 g/mol): H 2.00; N 9.33. Found: H 2.08; N 9.46. ^{1}H-NMR (DMF, 300.13 MHz): δ (ppm): 4.16 (broad s, 6H, NH_3).
